# Ivermectin: Potential Role as Repurposed Drug for COVID-19

**DOI:** 10.21315/mjms2020.27.4.15

**Published:** 2020-08-19

**Authors:** Alok Dixit, Ramakant Yadav, Amit Vikram Singh

**Affiliations:** 1 Department of Pharmacology, Uttar Pradesh University of Medical Sciences, Uttar Pradesh, India; 2 Department of Neurology, Uttar Pradesh University of Medical Sciences, Uttar Pradesh, India

**Keywords:** ivermectin, coronavirus disease 2019, SARS-CoV-2, drug repurposing, antiparasitic

## Abstract

Severe acute respiratory illness caused by 2019 novel coronavirus (2019-nCoV), officially named severe acute respiratory syndrome coronavirus (SARS-CoV-2) in late December 2019 is an extremely communicable disease. World Health Organization (WHO) declared coronavirus disease 2019 (COVID-19) as a pandemic as it has spread to at least 200 countries in a short span of time. Being a new disease there is lack of information about pathogenesis and proliferation pathways of this new coronavirus. Currently there is no effective treatment for coronavirus infection; major effort is to develop vaccine against the virus and development of therapeutic drugs for the disease. The development of genome-based vaccine and therapeutic antibodies require thorough testing for safety and will be available after some time. In the meanwhile, the available practical approach is to repurpose existing therapeutic agents, with proven safety record as a rapid response measure for the current pandemic. Here we discuss the presently used repurposed drugs for COVID-19 and the potential for ivermectin (IVM) to be used as a therapeutic option in COVID-19.

## Introduction

The severe acute respiratory syndrome caused by coronavirus 2 (SARS-CoV-2) has been detected as the pathogenic virus in the current outbreak which started in the city of Wuhan, Hubei Province of China in early December 2019. The Centre of Disease Control and Prevention in China, detected a new type of corona virus on 7th January 2020 causing coronavirus disease of 2019 (COVID-19), provisionally named as 2019 novel coronavirus (2019-nCoV) by World Health Organization (WHO) and later renamed as SARS-CoV-2 by International Committee on Taxonomy of Virus (1). SARS-CoV-2 is highly pathogenic virus, possesses mutative behaviour and causes rapid human to human spread. Specific treatment for COVID-19 has not been established yet, and the management of patients is primarily symptomatic or supportive care treatment. Anticipating that vaccine will take time and there is critical need of an effective treatment against COVID-19, many drugs are being repurposed for use among patients and healthcare workers for the purpose of prophylaxis and treatment of severe cases. These repurposed drugs include chloroquine phosphate, hydroxychloroquine (HCQ), lopinavir/ritonavir, umifenovir, remdesivir, favipiravir and monoclonal antibody tocilizumab.

## Repurposed Drugs for COVID-19

Chloroquine phosphate conventionally used for prophylaxis/treatment of malaria, and extraintestinal amoebiasis whereas hydroxychloroquine approved for suppressive treatment/treatment of acute attacks of malaria and rheumatoid arthritis are the two most commonly used drugs for COVID-19 worldwide. They predominantly act by increasing endosomal pH and interfere with the glycosylation of cellular receptor of SARS-CoV and thus it has the potential to block viral infection. Additionaly, chloroquine also inhibits the quinone reductase-2, which is involved in sialic acid biosynthesis (an acidic monosaccharides of cell transmembrane proteins required for ligand recognition) that makes this agent a broad antiviral agent (2). The mechanism of action of chloroquine and hydroxychloroquine are same, both act as a weak base that can change the pH of acidic intracellular organelles including endosomes/lysosomes, essential for the membrane fusion. Besides chloroquine also possesses immune-modulating properties which may augment the antiviral activity in vivo. It is believed that both the agents could be effective tools against SARS-CoV-1 and SARS-CoV-2 (3). Though they have shown promising activity against SARS-CoV-2, still there is a risk of arrhythmia associated with their administration at higher cumulative dosages; hence, vigilance is required with prior electrocardiogram for corrected QT (QTc) interval in vulnerable cases. Recent studies have reported contrasting results for the combination of hydroxychloroquine and azithromycin in COVID patients as regards viral load, positivity at day 7 and culture (4, 5).

Combination of lopinavir/ritonavir is used for the treatment of HIV and is a potential candidate for the treatment of COVID-19. The antiretroviral drug lopinavir, a protease inhibitor is formulated in combination with ritonavir which inhibits the metabolising enzyme cytochrome P450 3A and therefore increases the half-life of lopinavir (6). A randomised open labelled clinical trial of lopinavir/ritonavir 400 mg/100 mg, twice daily for 14 days (*n* = 99) has concluded that the combination was not more effective than standard care treatment and had similar recovery process in severe COVID-19 (7).

Remdesivir is a monophosphate prodrug that undergoes metabolism to an active C-adenosine nucleoside triphosphate analogue. Currently, remdesivir is a promising potential therapy for COVID-19 due to its broad-spectrum and potent in vitro activity against several novel coronavirus (nCoVs), including SARS-CoV-2 with EC_50_ (half maximal effective concentration) and EC_90_ (concentration to induce 90% maximal response) values of 0.77 μM and 1.76 μM, respectively (8).

Favipiravir is a prodrug of a purine nucleotide, favipiravir ribofuranosyl-5′ triphosphate and approved for therapeutic use in resistant cases of influenza. The active agent inhibits the ribonucleic acid (RNA) polymerase, inhibiting viral replication. In vitro, the EC_50_ of favipiravir against SARS-CoV-2 was 61.88 μM/L in Vero E6 cells (9).

## Ivermectin: An Antiparasitic Drug

Apart from above discussed drugs, an additional drug which has property to inhibit the replication of RNA viruses found in several studies is ivermectin (IVM). IVM, an anthelminthic drug is used to treat various parasitic infestations. This includes river blindness (onchocerchiasis), head lice, scabies, lymphatic filariasis, ascariasis, entrobiasis, strongyloidiasis and trichuriasis (10). IVM was discovered by Satoshi Omura of Kitasato University, Tokyo in 1975 and came to use in 1981. IVM was chemically derived from the avermectin family of compound, identified by William C. Omura from the bacterium *Streptomyces avermitilis* and purified by Campbell, has greater potency and lower toxicity (11). They act via binding to glutamate-activated chloride channels found in nematode nerve or muscle cells and causes hyperpolarisation by increasing intracellular chloride concentration, resulting in paralysis (12). The peak plasma concentration of IVM is achieved within 4 h–5 h after oral ingestion and is about 93% bound to plasma proteins. The drug is metabolised by hepatic microsomal enzymes CYP3A4 and adverse effect includes fever, pruritus, arthralgia, postural hypertension, tachycardia, oedema, lymphadenopathy, sore throat, cough and headache. IVM should be avoided in pregnancy and children below 5 years of age (13).

## IVM for Viral Diseases

Independent of its antiparasitic action, IVM has demonstrated broad spectrum antiviral property in vitro. IVM is shown to be effective in vitro against RNA and deoxyribonucleic acid (DNA) viruses, including human immunodeficiency virus-1 (HIV-1), dengue virus (DENV), influenza, Venezuelan equine encephalitis virus (VEEV) and Zika virus (14). Nuclear import of viral proteins plays important role in life cycle of many viruses, including RNA viruses that replicate in the cytoplasm such as DENV, respiratory syncytial virus (RSV) and rabies. IVM inhibits the interaction between integrase protein and importin α/β1-mediated nuclear import essential for HIV infection and was the earliest demonstration that inhibitors of nuclear import can have potent antiviral activity (15).

Pseudorabies virus (PRV) is the causative agent for pseudorabies (PR), which is an important swine disease. Virus can cause lifelong infection in pigs by residing in the trigeminal ganglia. Vaccines are used widely but are unable to provide absolute protection. Use of IVM, blocked the nuclear/nucleolar translocation of the accessory subunit of the DNA polymerase UL42 and reduced the lesions in mice caused by PRV infection in vitro and in vivo (16). This implies that IVM, a small-molecule inhibitor, can be a potential antiviral drug candidate against PRV infection. Likewise, VEEV, from the genus Alphavirus and family Togaviridae, can cause a fatal neurological disease in equines and humans. Endemic to northern South America and ranging into Mexico and the southern United States, the virus is transmitted between hosts and mosquitoes (11). Mifepristone and IVM were identified as importin alfa/beta1 inhibitors and useful in the treatment of VEEV (17).

IVM has been shown to inhibit nuclear import of host and viral proteins, including simian virus SV40 large tumour antigen (T-ag) and DENV nonstructural (NS1) protein 5. In DENV, a single daily oral dose of IVM has resulted in a significant reduction in serum level of viral NS1 protein, but no change in viral load has been observed (18). Infections with yellow fever virus (YFV), DENV, encephalitic viruses such as west nile virus (WNV), Japanese encephalitis (JEV) pathogenic flaviviruses create a serious global public health problem. IVM, by in-silico docking analysis, has been identified to inhibit the in vitro replication of different flaviviruses, specifically targeting NS3 helicase activity. In a cytopathic effect (CPE) reduction assay in Vero-B cell culture, the EC_50_ for inhibition of YFV replication was between 3.1 nM and 6.3 nM. IVM proved less active against DENV in the CPE reduction assay (EC_50_ 0.1 mM), although inhibition in virus yield reduction assays was observed (EC_50_ 0.7 mM). IVM has proved to be most potent against YFV and inhibit the in vitro replication of DENV, JEV and WNV, though less efficiently (19). Liposomes, microemulsion and polymeric micelles have been used to improve the pharmacological availability of IVM. IVM, when delivered through liposomes, reduced cytotoxicity up to five times, improves its activity and effectively inhibit DENV replication (20). Recently, the joint work of Biomedicine Discovery Institute, Monash University and the Peter Doherty Institute of Infection and Immunity has found that a single dose treatment effectively removed all SARS-CoV-2 viral RNA in a cell culture by 48 h. It is hypothesised that its action is likely through inhibiting IMP alfa/beta1-mediated nuclear import of viral proteins. Development of an effective antiviral for SARS-CoV-2, if given to patients early in infection, could help to limit the viral load, prevent severe disease progression and limit person-person transmission (18).

IVM is used in a dose of 0.15 mg/kg–0.2 mg/kg body weight for most of the parasitic infestations as oral tablet and is well tolerated. It will be reasonable to use similar dose empirically in COVID-19 patients with mild to moderate symptoms, PCR positive for SARS-CoV-2 virus, and without comorbidities on first encounter, till there is conclusive evidence from randomised controlled trials. Rarely adverse effects such as seizure, hypotension and worsening of asthma are observed with IVM and should be avoided in patients with history of allergy, liver disease and asthma. A combination of IVM (200 mcg/day once stat) with doxycycline (200 mg/day for 5 days) can also be attempted empirically as it has been administered previously for parasitic infections too.

COVID-19 pandemic has affected over 200 countries in a span of less than 4 months burdening the entire health care system with no viable drug which can inhibit the viral transmission or infectivity. Most of the drugs which are being used to treat this novel disease are not so effective and the development of vaccines is distant in near future, thus there is enormous requirement to repurpose the available drugs using concrete evidences. Present circumstances demand a therapeutically effective option against SARS-CoV-2 without delay. IVM which is a widely used as antiparasitic drug has shown to have antiviral activity in in vitro studies against HIV, dengue, influenza, VEEV and Zika virus. The broad-spectrum antiviral activity of IVM against both RNA and DNA viruses offers an added advantage to its potential use over other antiviral drugs. Studies are available for its use against RNA virus and have also been tested for its effectiveness against SARS-CoV-2 in vitro. Based on evidences it seems prudent to use IVM empirically and conduct well designed controlled randomised trials to prove its efficacy in vivo.
